# Impact of protein corona and light modulation on the antibacterial activity of light-activated silver nanoparticles†

**DOI:** 10.1039/d5tb00081e

**Published:** 2025-05-20

**Authors:** Varsha Godakhindi, Anjumana Jannati Nur, Mariya Munir, Juan L. Vivero-Escoto

**Affiliations:** a Department of Chemistry, University of North Carolina at Charlotte Charlotte NC 28223 USA jviveroe@charlotte.edu; b Nanoscale Science Program, University of North Carolina at Charlotte Charlotte NC 28223 USA; c Department of Civil and Environmental Engineering, University of North Carolina at Charlotte Charlotte NC 28223 USA; d Infrastructure and Environmental Systems Program, University of North Carolina at Charlotte, Charlotte, NC 28223 USA; e Center for Innovation, Translational Research and Applications of Nanostructured Systems, University of North Carolina at Charlotte Charlotte NC 28223 USA

## Abstract

Silver nanoparticles (AgNPs) as antimicrobial agents have gained extensive popularity due to their broad-spectrum action. Recently, AgNPs have been combined with photosensitizers (PS) to develop a synergistic antimicrobial effect. This synergy is associated with the light-activated increase in the release of Ag^+^, which drives the antibacterial mechanism against antibiotic-resistant bacteria (ARB). A factor typically not considered in the performance of AgNPs is the environmental conditions, such as salt and protein content, that significantly impact their bactericidal effect. In this work, we used protoporphyrin IX (PpIX) as a PS to synthesize PpIX–AgNPs. We elucidated the critical role of environmental conditions on the colloidal stability of PpIX–AgNPs in different bacterial culture media. We also determined the impact of the culture media on the light-activated release kinetics of Ag^+^. We found that cell media with lower protein and higher salt content drive the colloidal stability and release kinetics of Ag^+^ from AgNPs. Furthermore, we have shown that the multiple-irradiation approach of this light-controlled platform maximizes the release of Ag^+^ and promotes effective antibacterial action. We successfully tested this multiple-irradiation strategy in methicillin-resistant *Staphylococcus aureus* (MRSA) demonstrating ∼7-log-unit reduction at 1.5 μg mL^−1^ of PpIX–AgNP. A 6-log-unit MRSA inhibition was achieved in the nutrient broth (NB) media under the same irradiation strategy. We envision that this light-activated PpIX–AgNPs system can overcome major issues with the elimination of ARB and reduce side effects.

## Abstract 2

The critical role of environmental conditions on light-activated silver nanoparticles was elucidated. The multiple-irradiation approach of this platform maximizes the release of silver ions and promotes effective antibacterial action.

## Graphical abstract

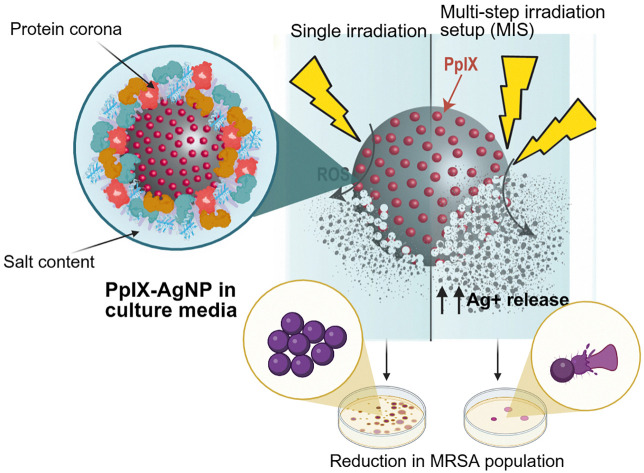

## Introduction

The rise of antibiotic-resistant bacteria (ARBs) has become one of the prevalent public health threats worldwide. In 2019, ARBs were directly responsible for 1.27 million deaths.^1,2^ The persistent failure of antibiotics and their pipeline crisis have compelled researchers to explore non-antibiotic alternatives. Nanomaterials have aroused a great interest in the field of antibacterials.^3^ Metal nanoparticles, nanozymes, and metal–organic complexes have been rationally designed and developed for antibacterial applications.^4–9^ Among them, silver nanoparticles (AgNPs) have shown promising outcomes due to their broad-spectrum antibacterial activity, generation of reactive oxygen species (ROS), disruption of the bacterial cell membrane, interference in the metabolic and DNA replication pathways, and denaturation of sulfur-containing proteins.^7,10,11^ The release of silver ions (Ag^+^) plays an essential role in contributing to the antibacterial activity of AgNPs.^12^ The Ag^+^ release from AgNPs can be regulated based on the physiochemical properties of nanoparticles (NPs), environmental conditions, and external triggers such as light.^12^ Among the environmental conditions, nutrient-rich medium, pH, or ionic strength affects the AgNPs colloidal stability and Ag^+^ release. In particular, proteins in bacterial medium tend to associate with the surface of NPs, forming a protein layer around it, a phenomenon termed “protein corona”.^13^ This NP–protein interaction is a dynamic process affecting the stability and biological identity of the NPs. It is governed by the physicochemical properties of NPs, such as size, morphology, and surface coating.^14^ In the case of AgNPs, several studies have also reported that protein corona impacts the release of silver ions and their colloidal stability, thereby hindering AgNP's antibacterial activity.^13,15–18^ Vasquez *et al*. detailed the role of various bacterial culture media on the stability of AgNPs and their subsequent antibacterial activity.^18^ The authors emphasized that the “chemical complexity” (diversity in the range of culture components) and composition of the bacterial medium can be correlated to the minimum inhibitory concentration (MIC) in the *E. coli* strain. However, a systematic investigation is needed to understand the correlation of Ag^+^ release to the culture conditions.

Light-activated silver nanoparticles as antimicrobials have been evaluated in multiple resistant bacterial and fungal species.^19–23^ Recently, some studies have reported the synergistic antibacterial effect of combining a photosensitizer (PS) with AgNPs (PS-AgNPs).^24–28^ PSs are molecules that absorb light at specific wavelengths, which later transfer that energy to oxygen to generate ROS.^29–31^ The studies assert that the antibacterial synergy of PS-AgNPs mainly originates from enhanced light-mediated ROS generation. However, only a few studies have shown that light-induced ROS also increases Ag^+^ release due to the oxidation of the AgNPs surface.^32,33^ Our group reported on the Ag^+^ release kinetic from light-activated AgNPs in aqueous media to understand the role of Ag^+^ in the antibacterial outcome.^34^ We concluded that the Ag^+^ release was more pronounced in solution with higher ionic strength than in nanopure water, resulting in up to 7–8 log units inactivation of methicillin-resistant *Staphylococcus aureus* strain (MRSA) and a wild-type multidrug-resistant (MDR) *E. coli*. This showed that environmental conditions could have a major impact on the bactericidal performance of PS-AgNPs; nevertheless, as far as we know, no reports of the effects of cell culture medium on the antibacterial behaviour of PS-AgNPs have been published. Another advantage of light-activable PS-AgNPs is that the light can be modulated on intensity, time, or irradiation cycles to maximize the generation of ROS and, as a direct result, enhance the Ag^+^ release efficiency. One study demonstrated that the continuous 30-minute laser irradiation of PS-AgNPs (Ce6-AgNP) results in a gradual Ag^+^ release. The authors showed that light can be modulated to control the Ag^+^ release with short laser pulses in an ON (5 min) and OFF (5–10 min) pattern. The release of Ag^+^ increased only during the ON stage, whereas the amount of Ag^+^ reached saturation during the OFF stage. This pattern repeats in subsequent irradiation cycles.^20^ Thus, modulating the number of light irradiation cycles of PS-AgNPs can be a promising strategy to control the release of Ag^+^ and maximize the bactericidal effect.

**Fig. 1:**
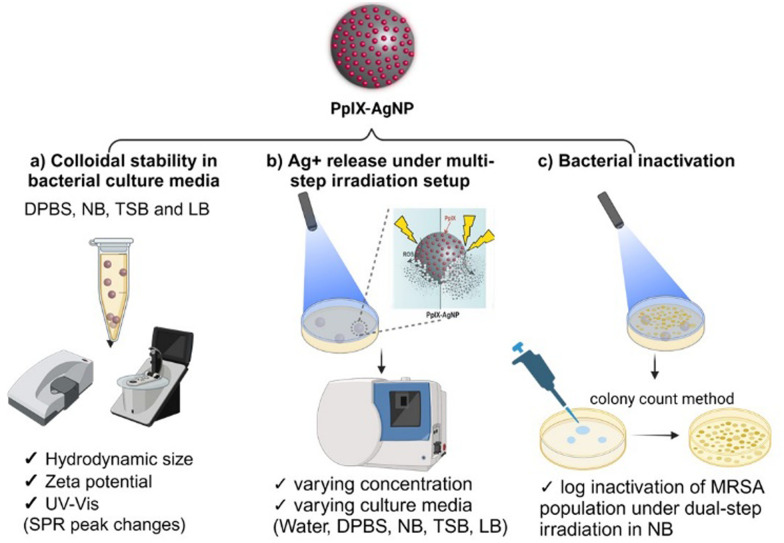
Schematic representation of the experiments carried out in this work. (a) Examine the colloidal stability of PpIX–AgNPs in bacterial culture media; (b) generate the Ag^+^ release under multi-step irradiation setup in various bacterial culture media; (c) evaluate the bacterial inactivation in MRSA under double-step irradiation condition in NB.

Herein, we used protoporphyrin IX (PpIX) as a PS that absorbs light in the visible range (400–700 nm) to synthesize PpIX–AgNPs. PpIX has been extensively used as an effective antimicrobial agent either as single molecule or attached to different materials.^35^ In this study, we assessed the role of culture media and light irradiation conditions affecting the antibacterial activity of PpIX–AgNPs (Fig. 1). We elucidated the critical role of the environmental conditions on the colloidal stability of PpIX–AgNPs in different bacterial culture media. Protein and salt content are major factors affecting colloidal stability and Ag^+^ release kinetics under variable culture media. We corroborated the hypothesis that light modulation in a multi-step irradiation setup of PpIX–AgNPs maximizes the Ag^+^ release. We also determined the impact of the culture media on the light-activated release kinetics of Ag^+^ under single and dual-step light irradiation setup. Finally, we tested the antibacterial action of PpIX–AgNPs under the dual-step irradiation strategy in MRSA. The antibacterial action of PpIX–AgNPs is attenuated by the cell media but can be compensated by the dual-step irradiation strategy to achieve ∼5-log reduction in MRSA population. We envision that the tunability of this light-activated PpIX–AgNP platform allows us to maximize its bactericidal performance with reduced amounts of the NPs.

## Materials

The following chemicals were purchased from the respective suppliers and used without modifications. Hydrochloric acid (Macron chemicals), nitric acid (Macron chemicals), protoporphyrin IX (Enzo Lifesciences), 1-ethyl-3-(3-dimethylaminopropyl) carbodiimide hydrochloride (EDC) (Oakwood Chemicals), tannic acid (95%, Acros Organics), dimethyl sulfoxide, dimethylformamide (VWR), dichloromethane (Alfa Aesar). Silver nitrate (AgNO_3_), silver standard for ICP, cysteamine hydrochloride, *N*-hydroxysuccinimide (NHS), dimethylamino pyridine (DMAP), *N*,*N*-diisopropylethylamine (DIPEA), trisodium citrate dihydrate were purchased from Sigma Aldrich. The Micro BCA™ Protein Assay Kit (23235) was purchased from Thermo Scientific.

The following bacterial broths were obtained from the respective suppliers: 1× Dulbecco's phosphate buffer saline (DPBS) (Corning), Luria Bertani (LB) broth (VWR), nutrient broth (NB) (Sigma-Aldrich) and tryptic soy broth (TSB) (BD Biosciences). All the broth cultures were autoclaved before use and stored at 4 °C.

## Experimental protocols

### Synthesis of PpIX–AgNPs

As reported previously, the protoporphyrin-conjugated silver nanoparticles (PpIX–AgNPs) were synthesized in a two-step process.^34^ In the first step, silver nanoparticles (AgNPs) were synthesized using the co-reduction method using sodium citrate and tannic acid.^36^ Briefly, a 100 mL aqueous solution of 5 mM trisodium citrate and 0.25 mM of tannic acid was brought to a boil at 100 °C under vigorous stirring. As the solution started boiling, a water condenser was used to prevent water loss, 1 mL of aqueous solution of 25 mM silver nitrate was added to the solution in one shot. The final solution was removed from heat when a color change was observed from colorless to yellow. The solution was cooled at room temperature and the AgNPs were centrifuged at 12 000 rpm for 15 min to remove excess tannic acid. These nanoparticles were washed three times with nanopure water under the same centrifugation conditions. The obtained AgNPs size was confirmed to be 42.2 ± 7.8 nm (*n* = 150) using TEM and was stored in nanopure water.

In the second step, a thiol-functionalized protoporphyrin derivative (cysPpIX) was synthesized using the previously reported protocol.^34,37^ The PpIX–AgNPs were obtained by reacting AgNPs with previously synthesized cysPpIX (1 : 0.5 wt) in dimethylformamide (DMF). Briefly, a solution of cysPpIX in DMF (1 mg mL^−1^) was prepared. AgNPs (1 mg) were centrifuged down at 12 000 rpm for 15 min to remove the water. Following this, 1 mL of DMF was added and sonication was used to disperse the AgNPs. This colloidal solution was transferred to a 20 mL scintillation glass vial and 500 μL of previously prepared cysPpIX solution in DMF was added. An additional 3.5 mL of DMF was added and the reaction mixture was vigorously stirred for 48 h at room temperature. After the reaction, the unreacted cysPpIX was collected *via* centrifugation at 12 000 rpm for 15 min. Nanoparticles were washed three times with DMF and separated under the same centrifugation conditions. The supernatant collected was analyzed using a UV-vis spectrophotometer to determine the amount of PpIX conjugated on the AgNPs surface. A calibration curve for absorbance at 500 nm and cysPpIX amount (mg) was generated. A typical batch fabricated under the above-mentioned conditions produced 1.2 ± 0.1 mg of PpIX–AgNPs. The synthesized PpIX–AgNPs were stored in DMF under dark conditions. The PpIX–AgNP concentrations are calculated in terms of AgNP and represented in the unit μg mL^−1^. For example, a concentration of 1.5 μg mL^−1^ of PpIX–AgNP is equivalent to 1.5 μg mL^−1^ of AgNP and 1 μM of cysPpIX.

### Characterization

Hydrodynamic size, polydispersity index (PdI) and zeta potential (*ζ*-potential) determination were performed using Malvern ZS series. The concentration used for DLS and *ζ*-potential measurements were fixed at 50 μg mL^−1^. UV-Vis spectra were obtained using Cary 50 UV-visible spectrophotometer (Varian). 3 mL of sample was used to obtain absorbance spectra from 200–800 nm. The corresponding culture media without nanoparticles were used as blanks and used to normalize the UV-vis spectra of PpIX–AgNPs in respective culture medium.

The size and morphology of the synthesized nanoparticles were analyzed using transmission electron microscopy (TEM, JEM 1230), operating at an accelerating voltage of 200 kV. The samples were prepared by dispersing AgNPs and PpIX–AgNPs in 50 μL of ethanol. Then, 10 μL of the sample was placed on the carbon-coated copper grid and air-dried for 1–2 h. The nanoparticle size and its distribution were calculated using ImageJ software. A sample size of 150 nanoparticles was considered for analysis, and the size was finally reported as average ± sd.

### Ag^+^ release profile using ICP-OES

The experimental setup was adapted from the previous reports.^34^ The release kinetics of Ag^+^ were assessed in two experimental irradiation setups: (a) single irradiation mode (b) multi irradiation mode (MIR), where the irradiation doses were varied in a step manner. For single irradiation setup, stock solutions of PpIX–AgNP in DMF equivalent to 150 μg mL^−1^ AgNPs concentration were prepared. The stock solution was diluted 100 times with the relevant media (DPBS, NB, TSB, LB) to obtain the final volume of 4 mL (final concentration = 1.5 μg mL^−1^). A Petri dish (60 × 15 mm) containing 4 mL of diluted solution (conc. = 1.5 μg mL^−1^) in relevant media was irradiated with a white light source (LumaCare LC-122 A, Fiber optic probe LUM V, 400–700 nm; 19 mW cm^−2^) for 20 min. Before irradiation, 100 μL sample was withdrawn indicating the time point as 0 min. Then, 100 μL was collected at specific time points from each sample at 5, 10, 20, 30 min, 1, 2, 4, 6, 12, 24 h. The collected samples were immediately centrifuged for 10 min at 12 000 rpm and the supernatant was collected for ICP-OES analysis.

In multiple irradiation setup, two sets of studies were conducted, the release profiles in varying culture media (final conc. = 1.5 μg mL^−1^) and for varying concentrations in DPBS. For these experiments PpIX–AgNPs stock in DMF corresponding to respective AgNPs concentrations of 50, 100, 150 μg mL^−1^ were prepared. Like single irradiation setup, the PpIX–AgNP stock were diluted 100 times in relevant media to obtain final concentration of 0.5, 1 and 1.5 μg mL^−1^ and transferred to Petri dish. The samples are then irradiated with the white light source for 20 min, followed by 40 min of incubation at room temperature in dark conditions. This sequence (1 h duration = 20 min irradiation + 40 min resting period) was termed as one irradiation step/cycle. At the end of the 40 min incubation, the sample was irradiated again following the same sequence, constituting as the 2nd irradiation step. Before irradiation, 100 μL sample was withdrawn indicating the time point as 0 min. During each irradiation step, 100 μL of the aliquots were collected at time points, 10, 20, 30, 45 and 60 min. The collected samples were immediately centrifuged for 10 min at 12 000 rpm and the supernatant was collected for ICP-OES analysis.

To determine the total amount of Ag^+^ in all samples, aliquots of the nanoparticles were digested by performing a “cold digestion” followed by “hot digestion”.^38^ The digestion protocol was optimized and modified in terms of time and volume. Cold digestion included mixing 50 μg of NPs with 3.0 mL of concentrated HNO_3_ and incubating at room temperature for 30 min. Later, this mixture was heated under 150 °C using an oil bath for 4–6 h to allow excess HNO_3_ to evaporate (hot digestion). The remaining volume was measured using a graduated cylinder and diluted using 2% HNO_3_. The samples were filtered through a 0.22 μm polyethersulfone (PES) membrane before ICP-OES analysis. A calibration curve was obtained using a silver standard diluted in 2% HNO_3_ to prepare standard concentrations of 10, 25, 50, and 100 μg L^−1^. A new set of calibration curves were generated for each run. The total amount of Ag^+^ digested (in μg) was calculated using the calibration curve and expressed in terms of Ag^+^ amount per μg of NP (%) (Table S1, ESI†). The concentration of PpIX–AgNPs (1.5, 1.0, 0.5 μg mL^−1^) used for release were digested using the protocol and the digestion results were expressed in μg L^−1^ (Table S4, ESI†). These values were to calculate % of Ag^+^ release using the following equation,%Ag^+^ release = Ag^+^ released at time point “*t*” (μg L^−1^)/total Ag^+^ digested (μg L^−1^)

The kinetic release rates were determined by performing linear fit on the Ag^+^ release kinetic plots using OriginPro 2024 (Student version). The *R*^2^ and slope values corresponding to burst and slow release were recorded.

The Ag^+^ release was quantified using inductively coupled plasma – optical emission spectroscopy (ICP-OES) (Optima 3000, Agilent). Samples obtained from post digestion and release experiments were introduced into plasma *via* a peristaltic pump and discharged as an aerosol suspended in argon gas. The data acquisition was performed in triplicates with the torch assembly in the axial mode. The default acquisition parameters used are RF = 1.2 kW; auxiliary gas flow = 1 L min^−1^, nebulizer gas flow = 0.7 L min^−1^; plasma flow = 12 L min^−1^, pump speed = 12 rpm; stabilization time = 15 s; sample uptake time = 25 s; rinse time = 30 s and Ag analytical line = 328.068 nm.

### Bacterial inactivation experiment

#### Preparation of bacterial strain

Methicillin-resistant *Staphylococcus aureus* (MRSA) strain BAA-44, purchased from ATCC, was used as the test organism. LB agar plates were used to subculture the MRSA strain. A single colony from the LB agar plate was inoculated in LB broth and incubated at 37 °C for ∼18 h, resulting in actively growing cells. The fresh cultures were centrifuged at 7000 rpm for 5 minutes the following day, and the collected cells were washed twice with 1× DPBS to remove any residual particles from the broth. The washed cells were resuspended in relevant media (1× DPBS and NB) to achieve optical density (O.D) at 600 nm corresponding to 0.5 McFarland standard, *i.e.* ∼1.5 × 10^8^ CFU per mL. For NB experiments, the O.D lower than 0.5 McFarland standard was considered, to achieve reduced initial bacterial load (∼10^7^ CFU per mL).

#### Irradiation of bacterial cells

Before exposure to light, the bacterial suspension in respective culture media was incubated in the dark at room temperature for 30 minutes with varying concentrations of AgNPs and PpIX–AgNPs. To achieve the working concentrations 1.5 μg mL^−1^, 1 μg mL^−1^, and 0.5 μg mL^−1^ from a stock of 150 μg mL^−1^ AgNPs and PpIX–AgNPs, the bacterial and nanoparticles were prepared by mixing 3960 μL of bacterial suspension with 40 μL of stock sample, 3973 μL of bacterial suspension with 27 μL of stock sample, and 3986 μL of bacterial suspension with 14 μL of the stock sample respectively. The suspensions were exposed to the white light source for 20 min for the single irradiation experiment. For the multiple irradiation experiment, the samples were incubated for 40 min in the dark at room temperature after the initial light exposure, and then subjected to a second irradiation for another 20 min. All the experimental sets were conducted in triplicates with negative and dark controls.

#### Enumeration of surviving bacterial cells

The survival of the bacterial cells was determined at 3 different time points- immediately after irradiation (0 h), 4 h and 24 h post irradiation in duplicate using the drop plate method where 100 μL of samples from all experimental sets were diluted in 900 μL of respective diluent. Subsequently, 20 μL of each dilution was spotted on LB agar plate. The log inactivation was calculated using eqn (1) which relates the initial and final concentrations of bacterial cells.

1 log inactivation of bacteria = log(*C*_o_/*C*_*t*_)

Here, *C*_o_ = concentration (CFU per mL) of bacteria without the addition of nanoparticles and *C*_*t*_ = bacterial concentration after the addition of nanoparticles and (or) light irradiation after time *t*.

#### Statistical analysis

Graphs and statistical analyses were performed using OriginPro 2024 (Academic Version). Statistical significance between each irradiation strategy was assessed by one-way analysis of variance (ANOVA), with the Tukey test performed for mean comparison. All the statistical analyses were performed using OriginPro 2024 (Academic Version) with *α* = 0.05 and reported as stars assigned to the *p*-values. The exact *p* values are in the experiment's main paper.

## Results and discussion

### Synthesis and characterization of PpIX–AgNPs

The TEM size of AgNPs obtained was 42.2 ± 7.8 nm (*n* = 150). The AgNPs were reacted with cysteamine-modified PpIX (cysPpIX) in DMF based on our previously published work.^34^ AgNPs were synthesized based on the co-reduction of silver nitrate (AgNO_3_) using sodium citrate and tannic acid.^36^ The cysPpIX derivative was synthesized in a two-step approach; first, the carboxylic groups were activated using NHS, affording an ester group to obtain succinimide ester PpIX (sePpIX). As a second step, a nucleophilic acyl substitution was carried out with cysteamine to obtain the final cysPpIX.^37^ The successful synthesis of sePpIX and cysPpIX derivatives was confirmed using UV-vis, FT-IR, and MALDI (Fig. S1, ESI†).

**Fig. 2:**
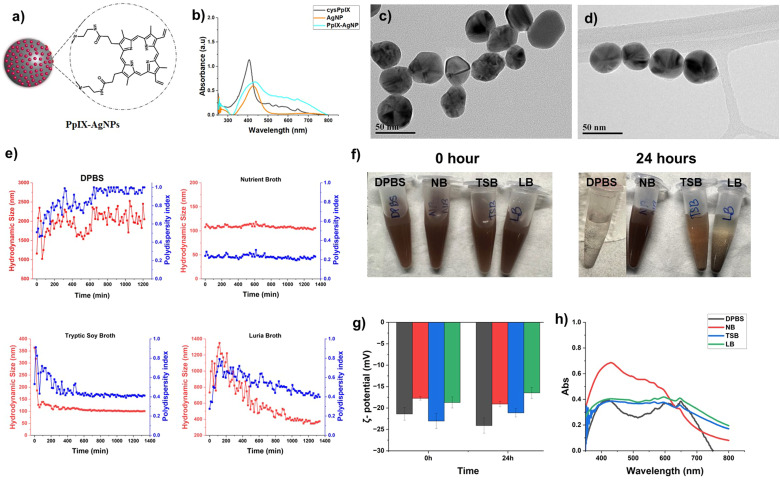
(a) Schematic representation of PpIX–AgNPs. (b) UV-Vis absorption spectrum for cysPpIX, AgNPs and PpIX–AgNPs. TEM images of (c) AgNPs and (d) PpIX–AgNPs. Colloidal stability of PpIX–AgNPs in DPBS and bacterial culture media, nutrient broth (NB), tryptic soy broth (TSB), and Luria Bertani broth (LB). (e) Hydrodynamic size (red) and polydispersity (blue) change over 24 h for PpIX–AgNPs in DPBS, NB, TSB, and LB. (f) Visual images of PpIX–AgNP in bacterial culture media at 0 and 24 h. (g) Effect of bacterial culture media on *ζ*-potential values. (h) UV-Vis absorption spectrum for PpIX–AgNPs in bacterial culture media.

The cysPpIX obtained was further reacted with AgNPs in DMF in the ratio of 0.5 : 1.0 wt (cysPpIX : AgNP) to obtain the final material, PpIX–AgNPs (Fig. 2a). Unlike physical interactions between PSs and AgNPs, chemical conjugation ensures stable nano-formulations with minimal leakage. A chemical thiol-silver bond between PS and AgNP enables precise control of distance and targeted delivery, enhancing antibacterial synergy.^39^ These NPs were characterized by UV-vis spectroscopy to determine the PpIX content, *ζ*-potential, and dynamic light scattering (DLS). The UV-vis of AgNPs is generally characterized by an absorption peak around 420–425 nm, associated with the surface plasmon resonance (SPR) peak of AgNPs. As seen in Fig. 2b, the UV-vis spectrum for AgNPs depicts the characteristic SPR absorption band. In the case of PpIX–AgNPs, the UV-vis spectrum shows a strong absorption band in the 425–435 nm range related to the Soret- and SPR-band of PpIX and AgNPs, respectively. However, unlike AgNPs, PpIX–AgNPs shows additional absorption bands in the region of 500–700 nm associated with the Q-bands of PpIX (Fig. 2b). These results confirm the successful conjugation of PpIX molecules to AgNPs. To quantify the amount of PpIX loaded to AgNPs, the post-reaction supernatant and washing solutions were collected. The absorbance at 500 nm was measured and using a calibration curve the % loading of PpIX on AgNPs was quantified (Fig. S3, ESI†). The percentage was determined to be 44.8 ± 0.3 wt% (*n* = 6). The PpIX molecules functionalized per AgNP was calculated to be 241 831, as per previously reported work.^34,40^ The detailed calculations is provided in ESI.† Furthermore, TEM images indicated that AgNPs and PpIX–AgNPs have spherical morphology with diameters of 42.2 ± 7.8 and 42.2 ± 8.9 nm (*n* = 150), respectively (Fig. 2c, d and Fig. S2, ESI†). As indicated in Table S1 (ESI†), the hydrodynamic size measured by DLS indicated a slight increase from 37.8 ± 0.3 nm (AgNP) to 64.1 ± 0.6 nm (PpIX–AgNP) in DPBS (1 mM), which is due to aggregation of nanoparticles as an indication of the presence of PpIX on the surface of AgNPs. The *ζ*-potential also showed a slight increase in negative charge from −47.1 ± 3.6 (AgNP) to −56.7 ± 3.0 (PpIX–AgNP).

### Study of the colloidal stability of PpIX–AgNP in different bacterial culture conditions

The colloidal stability of the PpIX–AgNPs was studied in different cell culture media including DPBS (10 mM), Luria Bertani (LB) broth, nutrient broth (NB) and tryptic soy broth (TSB). The following properties were determined as indicators of colloidal stability: hydrodynamic diameter (*D*_h_), polydispersity index (PdI), *ζ*-potential, and UV-vis spectroscopy. The *D*_h_ and *ζ*-potential of PpIX–AgNPs were monitored at the concentration of 50 μg mL^−1^. The changes in *D*_h_ of the PpIX–AgNP were evaluated from 0–24 h in the respective culture media (Fig. 2e). The initial *D*_h_ size at 0 h was observed to be lowest in NB (107.8 ± 0.5 nm), followed by TSB (385.2 ± 3.4 nm), LB (772.5 ± 29.7 nm) and finally DPBS (816.1 ± 76.3 nm). PpIX–AgNPs in NB show excellent colloidal stability throughout the whole stability evaluation, with *D*_h_ varying in the 100–120 nm range and PdI remaining consistent at 0.2. However, in the case of TSB, and LB, the *D*_h_ shows a significantly higher value at 0 h, which eventually drops from ∼385 nm to ∼150 nm in TSB and ∼1350 nm to ∼350 nm in LB. It should be noted that the drop in *D*_h_ is faster in TSB, within 60 min; whereas in LB, it is a gradual drop that happens over 16–17 h. In the case of DPBS, the DLS results indicates poor colloidal stability, as demonstrated by the constant fluctuation in *D*_h_ (1000–2000 nm) with a the PdI value reaching as high as 1.0. As seen in Fig. 2f, PpIX–AgNPs in all media except NB shows visible precipitation after 24 h of incubation. Interestingly, a similar trend in the colloidal stability of PpIX–AgNPs as the one with NB was observed in water where *D*_h_ remains consistent throughout the 24 h duration at around ∼100 nm, indicating good colloidal stability, supported by a PdI = 0.2 (Fig. S4, ESI†). It is also well-established that the protein corona reduces the surface charge.^41^ The changes in the net surface charge of nanoparticles are usually a great indicator of their colloidal stability since the electrophoretic mobility of nanoparticles in a solution is driven by surface charge.^42^ As seen in Fig. 2g and Table S2 (ESI†), at the initial time, the *ζ*-potential value was −21.4 ± 1.5 mV in DPBS, −17.8 ± 0.4 mV in NB, −23.1 ± 1.8 mV in TSB, and −18.7 ± 1.3 mV in LB. Post 24 h incubation, there are only minor changes in *ζ*-potential in DPBS (3–5 mV), while the culture media show only a minuscule difference of 1–2 mV.

UV-Vis spectroscopy is also a good indicator of colloidal stability for metal nanoparticles such as gold and silver NPs because the shift in SPR peak is associated with AgNPs surface coating, surrounding environment, and NP plasmonic coupling.^43–45^ Redshift is related to a shift in the SPR peak at longer wavelengths, whereas blueshift is associated with a shift in the peak to shorter wavelengths. Blueshift is linked to a decrease in the NP size,^46^ and redshift corresponds to changes in NP surface coating or aggregation.^44,47^ We measured the UV-vis spectrum for PpIX–AgNPs in different conditions: water, DPBS (10 mM), NB, TSB, and LB. As seen in Fig. 2h and Fig. S4 (ESI†), the UV-vis spectra in water and DPBS indicate that SPR peak maximum wavelength is observed at 420 nm. However, this SPR peak underwent red shift in all the cell media conditions with maximum wavelengths at 429, 432, and 433 nm for NB, TSB and LB, respectively. As expected, a red shift is observed in comparison with DPBS and water, which can be associated with the interaction of cell media protein with the surface of AgNPs, which is known as protein corona. This was confirmed using a colorimetric BCA assay (Pierce™ BCA Protein Kit). This kit was used to qualitatively compare the protein content bound to the NP post-exposure to the variable media. The protein content of PpIX–AgNPs in the cell media was qualitatively evaluated using BCA assay (620 nm). PpIX–AgNPs were dispersed in media for 24 h and washed 3 times, the BCA assay was performed on the final NPs as per kit instructions. As seen in Fig. S5 (ESI†), PpIX–AgNPs in TSB show the highest protein corona followed by LB and NB. These results follow a similar trend on the actual protein content in the cell media (Table S3, ESI†).

To thoroughly analyze the influence of bacterial culture medium on the colloidal stability of PpIX–AgNPs, it is essential to analyze the composition of each media formulation to account for protein and salt content (w/v%; Tables S3 and S4, ESI†). Based on the long-term colloidal stability analysis described above (Fig. 2e), PpIX–AgNPs have the highest colloidal stability in NB, while the colloidal stability deteriorates in DPBS, TSB, and LB. These trends in colloidal stability performance, with NB > TSB > LB > DPBS, could be attributed to the presence of salt content: NB (0.6%) < TSB (0.8%) < LB (1%) ∼ DPBS (0.95%). The amount of salt content drives the colloidal stability performance of PpIX–AgNPs, with NB having the lowest salt content but showing the highest colloidal stability for the NPs. This behaviour also correlates to the precipitation observed in NB and TSB (Fig. 2f). On the contrary, DPBS and LB display variable stability despite containing similar salt content. This can be attributed to the protein content in LB (1.5%) that may render colloidal stability to the NPs despite the high salt content (1%) (Table S4, ESI†). It should be noted that NB has a protein content of 1.8% w/v, which also contributes to its superior colloidal stability. Our results show that NB is the cell media with the best colloidal stability behaviour for PpIX–AgNPs. Salt content plays a dominant role in driving colloidal stability, but the protein content can also impact it. These findings support the previous studies expanding on the role of bacterial culture media on AgNP colloidal stability.^18^

### Investigation of Ag^+^ release profile under multi-step irradiation setup (MIS)

The PS attached to AgNPs under light irradiation is known to enhance the generation of Ag^+^ by oxidizing the AgNPs' surface.^33,48^ This process is mediated by ROS generated by the PS during the light irradiation. Our group previously demonstrated that the light-activated Ag^+^ release kinetics after single irradiation are composed of a burst and steady phase.^34^ The burst phase was associated with the irradiation time, followed by a steady phase representing the long-term passive release of Ag^+^. Our study showed that the media used for release has a significant impact; the ionic composition of DPBS drastically favors Ag^+^ release compared with nanopure water. In this work, we hypothesized that the release of Ag^+^ can be further enhanced by applying multiple irradiation cycles.

**Fig. 3:**
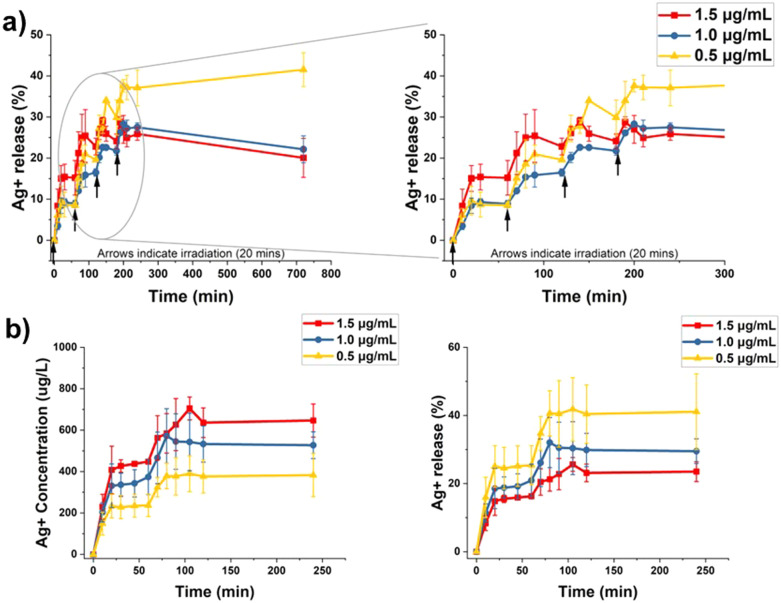
Ag^+^ release kinetics for varying concentrations of PpIX–AgNPs tested in (a) multi-step irradiation (MIS) and (b) dual-step irradiation strategy – cumulative Ag^+^ concentration and Ag^+^ release efficiency (%). The arrows indicate the start of a MIS irradiation.

Each irradiation cycle for the multiple irradiation setup (MIS) is composed of a 20 min round of irradiation with white light (400–700 nm, 19 mW cm^−2^), followed by 40 min of resting period in the absence of light at room temperature for a total of 60 min (Scheme S1, ESI†). The PpIX–AgNPs concentrations are represented in terms of AgNPs concentrations in this entire study. We tested the MIS experiment at different concentrations of PpIX–AgNPs (1.5, 1.0, and 0.5 μg mL^−1^) in DPBS using 4 cycles of irradiation. The amount of Ag^+^ released was measured by ICP-OES at different time intervals. During each cycle, aliquots were collected at time points; 0, 10, 20, 30, 45, and 60 min. The beginning of each cycle is indicated by an arrow in Fig. 3a. A final aliquot was collected 12 h after the first cycle. In general, the Ag^+^ release profiles resemble a step function with the increase in Ag^+^ release corresponding to the irradiation window (burst release = 20 min) and indicating no release after around 30 min (steady phase) (Fig. 3a and Fig. S6, ESI†). This behavior was observed for all the concentrations. It is also important to note that at 1.5 μg mL^−1^ concentration, ∼700 μg L^−1^ of the cumulative Ag^+^ release was achieved within the first two irradiation cycles (Fig. S6, ESI†). Moreover, the Ag^+^ release remains saturated for the same concentration, implying that the 3rd and 4th irradiation cycle provides minimal improvement in Ag^+^ release. Hence, the two cycles of irradiation (dual-step irradiation) suffice to achieve a higher cumulative Ag^+^ release. This irradiation setup can easily be implemented in bacterial inactivation experiments.

The dual-step irradiation strategy was recorded for 4 h (Fig. 3b). The maximum cumulative release of Ag^+^ at the end of the experiment follows the expected concentration-dependent trend with 1.5 > 1.0 > 0.5 μg mL^−1^. However, like the MIS experiments, the 0.5 μg mL^−1^ showed the highest percentage of Ag^+^ released from the initial amount at 40%, followed by 1.0 μg mL^−1^ (30%) and 1.5 μg mL^−1^ (25%). The percentage of Ag^+^ release for each concentration was determined by considering the total amount of Ag^+^ as the one obtained by a digestion protocol (Table S5, ESI†). The release rates for the two burst phases (B1 and B2) were calculated using linear curve fitting (Fig. S7 and Table S6, ESI†). The release rate (B1) gradually decreases with decreasing concentration, *i.e.*, 1.5 μg mL^−1^ (21.50 μg L^−1^ min^−1^) > 1.0 μg mL^−1^ (18.68 μg L^−1^ min^−1^) > 0.5 μg mL^−1^ (12.31 μg L^−1^ min^−1^). It is interesting to note that the release rates in burst phase 2 (B2) are similar for all concentrations (7–10 μg L^−1^ min^−1^). Overall, the dual-step irradiation strategy can be applied to accelerate Ag^+^ release in a controlled fashion. Moreover, we anticipate that this approach will maximize the release of Ag^+^ in conditions with lower dosages of PpIX–AgNPs.

### Investigation of Ag^+^ release profile in varying bacterial culture media

**Fig. 4:**
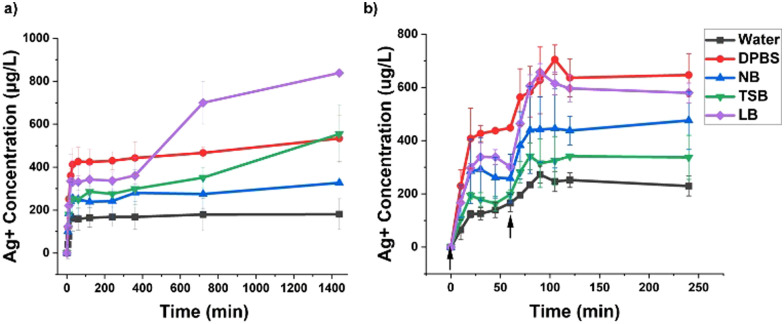
Ag^+^ release kinetic for PpIX–AgNPs (1.5 μg mL^−1^) in bacterial culture media under (a) single irradiation setup and (b) dual-step irradiation setup. The arrows indicate the start of a MIS irradiation cycle (irradiation time = 20 min).

Environmental conditions play a vital role in Ag^+^ release from AgNPs. Previous studies have reported that cell media composition influences Ag^+^ release.^18,49,50^ As far as we know, no study has reported the effect of media composition on Ag^+^ release kinetics, especially for light-activated AgNPs. In this study, we investigate the impact of different culture media including NB, TSB and LB, on the release of Ag^+^ after a single and dual-step light irradiation. DPBS and water were considered as control experiments, as reported previously.^34^ The study was tested for 24 h using a concentration of 1.5 μg mL^−1^ of PpIX–AgNPs. As seen in Fig. 4, in a single irradiation mode, a burst and a steady release phase were observed in all media conditions. In the burst phase, the cumulative Ag^+^ release shows trends as DPBS > LB > TSB > NB > water. The release rate was calculated using a linear curve fitting, as indicated in Fig. S8 and Table S7 (ESI†). The highest release rate was obtained for DPBS (23.66 μg L^−1^ min^−1^), followed by LB (22.21 μg L^−1^ min^−1^), NB (17.83 μg L^−1^ min^−1^), TSB (12.34 μg L^−1^ min^−1^), and finally water showing slowest release rate (8.26 μg L^−1^ min^−1^). We have previously demonstrated that the ionic composition in the media drastically favors Ag^+^ release; nevertheless, in these experiments, the other components in the bacterial culture media also affect the Ag^+^ release. The higher salt content (Table S4, ESI†) in DPBS (0.95%) and LB (1%) drive the release of Ag^+^, resulting in concentrations of ∼361 μg L^−1^ and 334 μg L^−1^, respectively, in the first 20 min of irradiation. The lower release in TSB, NB, and water is due to lower salt content. We have shown in our previous work that the presence of ions such as chlorides have a major impact on the dissolution of Ag^+^, which enhances the release of Ag^+^ from AgNPs.^34^ It is also known that protein content also plays a crucial role in Ag^+^ release. Unlike the salt content, the higher protein content in TSB and NB may also hinder the Ag^+^ release. As reported in the literature, protein corona can prevent the generation of ROS and release of Ag^+^ by blocking the interaction of environment with nanoparticles.^18^ In the case of TSB, higher protein content (2%) results in the lowest release rate (12.34 μg L^−1^ min^−1^), resulting in an Ag^+^ concentration of 248 μg L^−1^ in the burst phase. Our results confirm that NB, which has the intermediate protein (1.8%) and lowest salt content (0.6%), shows the lowest Ag^+^ concentration (240 μg L^−1^) among the culture media. Water, lacking salt and protein content, shows the lowest Ag^+^ release (170 μg L^−1^) for all the conditions tested. It has been noted in the literature that the presence of NaCl can promote the release of Ag^+^, whereas the presence of protein can passivate the surface of AgNPs, inhibiting the Ag^+^ release.^15,18,51,52^ Other studies have reported similar trends on the role of culture media in the release of Ag^+^.^15,50^

The impact of the media on the final amount released of Ag^+^ was different with the following trend: LB > DPBS ∼ TSB > NB > water (Fig. 4a). The Ag^+^ release was superior in LB media (838 μg L^−1^), followed by TSB (554 μg L^−1^) and DPBS (534 μg L^−1^). At the 24-hour timepoint, Ag^+^ release in LB was significantly higher than water (*p* = 0.0007), DPBS (*p* = 0.04), NB (*p* = 0.002), and TSB (*p* = 0.02), as seen in Fig. S8 (ESI†). Additionally, culture media in TSB and LB showed increased Ag^+^ release with kinetic rates of 0.14 μg L^−1^ min^−1^ and 0.39 μg L^−1^ min^−1^, respectively in the burst phase. Both media also show a sharp increase in Ag^+^ release post-6-hour time point. LB shows the highest concentration of Ag^+^ (838 μg L^−1^), most likely due to the high salt content (1%), particularly the presence of NaCl. Similarly, the increased performance in the TSB relative to the burst phase can be attributed to intermediate salt content (0.8%), which drives the increase in Ag^+^ concentration (554 μg L^−1^) in the steady phase. NB shows a passive release rate of Ag^+^ at 0.06 μg L^−1^ min^−1^ and final concentration of 326 μg L^−1^, which is the lowest overall release observed in cell media. The higher protein and lower salt content contribute to this behavior. A dark control experiment (Fig. S9, ESI†) shows a minimal Ag^+^ release for NB and TSB compared to the light-irradiated counterpart. However, LB under dark conditions also shows an increase in Ag^+^ release in the steady phase that can be attributed to the higher salt content. Previous reports support the claim that salt (NaCl) plays an essential role in Ag^+^ release in LB (PEG functionalized AgNP). However, other components such as yeast extract and tryptone also contribute to Ag^+^ release.^49^ This increased interaction of Ag^+^ ions with oxygen-, nitrogen-, or sulfur-containing components such as tryptone and glucose may drive more Ag^+^ ions into the culture media *via* Le Chatelier's principle.^53^

The dual-step irradiation strategy was determined to be optimal in DPBS, here we tested the same strategy in cell media. In this approach, there are two burst phases, B1 and B2, which correspond to two cycles of irradiation, indicated by the arrows in Fig. 4b. The Ag^+^ release profiles were determined at an initial concentration of PpIX–AgNPs of 1.5 μg mL^−1^ during 4 h. The results showed that a higher release was achieved in the dual-step mode than the single irradiation approach during the same time frame (Fig. S10, ESI†). For example, under a single irradiation setup in NB, the max Ag^+^ release of 327 μg L^−1^ was achieved, whereas, in the dual-step mode, the maximum Ag^+^ release achieved is 476 μg L^−1^ within 4 h. These results varied in different media conditions, with maximum cumulative Ag^+^ release at 4 h following the trend, DPBS ∼ LB > NB > TSB > water (Fig. 4b). Under dual-step approach, DPBS still shows the highest Ag^+^ release of ∼646 μg L^−1^ with release rates of 9.93 μg L^−1^ min^−1^ (B1) and 6.61 μg L^−1^ min^−1^ (B2). For most media conditions, the B1 and B2 release rates were similar (Table S8 and Fig. S11, ESI†), except for NB with values of 12.59 μg L^−1^ min^−1^ (B1) and 7.79 μg L^−1^ min^−1^ (B2), which are even higher than DPBS. Considering that the release of Ag^+^ was the lowest (242 μg L^−1^) in NB under single irradiation at 4 h, the dual-step irradiation strategy made a major impact increasing almost 100% the release of Ag^+^ (475 μg L^−1^) within the same period. This points to the advantage of using such a strategy for overcoming limitations related to hindered Ag^+^ release in single irradiation. Therefore, the enhanced Ag^+^ release using the dual-step irradiation strategy together with the colloidal stability of PpIX–AgNPs, make NB the best culture medium to evaluate the antibacterial activity of PpIX–AgNPs.

### Bacterial inactivation experiment

**Fig. 5:**
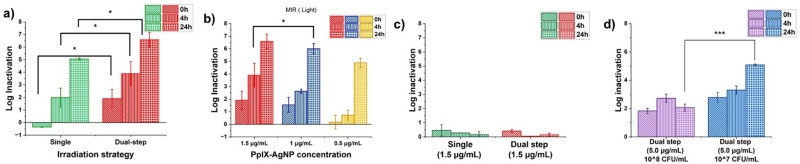
Antibacterial activity assessed in MRSA under variable light irradiation and culture conditions. For single irradiation, the timepoint 0 h indicates the inactivation immediately post-irradiation, whereas 4 h and 24 hours correspond times post-irradiation. Similarly, for dual-step, 0 h indicates the inactivation achieved after dual-step irradiation (immediately after 2nd irradiation). The other two time points, 4 and 24 h, show the antibacterial effect post-dual-step irradiation. The error bar is the standard deviation of three independent experimental replicates. (a) The antibacterial activity of 1.5 μg mL^−1^ PpIX–AgNPs in DPBS tested in single (green) and dual-step (red) irradiation setup. (b) The antibacterial activity of varying concentrations of PpIX–AgNP in DPBS tested in a dual-step irradiation setup. (c) The antibacterial activity of varying concentrations of PpIX–AgNP in NB. All the statistical analyses were performed with one-way ANOVA (with Tukey mean comparison) at *α* = 0.05 and reported as stars assigned to the *p*-values: ***p ≤ 0.0001, **p ≤ 0.001, *p ≤ 0.05, and ns *p* > 0.05.

MRSA is one of the most common causes of infections and is part of the ‘nine bacteria of international concern’;^54–58^ therefore, efficient alternatives to eliminate MRSA are needed. We have shown that PpIX–AgNPs can be used for the efficient light-activated nanomaterial to eliminate MRSA in DPBS under a single irradiation cycle.^34^ Here, we evaluated the antibacterial performance of PpIX–AgNPs under single and dual-step irradiation setups in DPBS and NB. In the case of dual-step setup, each irradiation cycle comprises 20 min irradiation of white light (400–700 nm, 19 mW cm^−2^), followed by 40 min of a resting period in the absence of light at room temperature before the next cycle (Scheme S1, ESI†). PpIX–AgNPs were used at a concentration equivalent to 1.5 μg mL^−1^ in terms of AgNPs or 1 μM in terms of cysPpIX against MRSA (BAA-44 strain from ATCC). The PpIX–AgNPs concentrations are represented in terms of AgNPs concentrations in the entire study. The bacterial log inactivation was determined by using the colony count method by collecting samples immediately after 0, 4, and 24 h post-irradiation.^34^ As seen in Fig. 5a, the dual-step irradiation achieved a significantly better log of inactivation in the MRSA population in DPBS than the single irradiation (*p*-value <0.05). At the 24 h post-irradiation timepoint, incorporating a dual-step irradiation setup increased the log of inactivation of MRSA from 5.0 to 6.6 log reduction, which corresponds to about 30 times higher MRSA reduction than the single irradiation approach (*p* = 0.04). Similar improvement was observed at 0 and 4 h, corroborating the advantage of using the dual-step-irradiation strategy to efficiently eliminate MRSA. Control experiments were performed using an equivalent amount of AgNPs (1.5 μg mL^−1^) and cysPpIX (1 μM) concentrations under both light and dark conditions. Fig. S12 (ESI†) shows that both AgNPs and PpIX–AgNPs achieved <1 log inactivation at 1.5 μg mL^−1^ concentration AgNP concentration (or 1 μM cysPpIX concentration) under dark conditions regardless of the irradiation set-up and incubation period. AgNPs achieved 2.17 log inactivation after 24 h post-irradiation, which is negligible compared to the inactivation achieved by PpIX–AgNPs as described above. AgNPs are known to under localized surface plasmon resonance (LSPR) under visible light excitation causing localized surface temperature increase, near field enhancement surrounding the NP and generation of ROS.^59^ The light excitation enhances the ROS generation *via* plasmon-assisted hot-carriers such as electrons and their subsequent reaction with oxygen in the media.^60–62^ The control experiment with cysPpIX under dark conditions does not afford any inhibition effect (Fig. S12, ESI†). However, upon light exposure for 20 min, the results showed 3 logs of inactivation of MRSA, which is expected due to the spontaneous generation of ROS by the cysPpIX. Nevertheless, PpIX–AgNPs have better antibacterial performance against MRSA than AgNPs and cysPpIX alone.

To evaluate the relevance of using the dual-step irradiation method to optimize the amount of PpIX–AgNPs needed for bacterial inactivation of MRSA, a set of experiments were carried out in DPBS using different concentrations of PpIX–AgNPs including 1.5, 1.0, and 0.5 μg mL^−1^ (Fig. 5b). We hypothesized that the dual-step irradiation setup would allow the optimization of the initial concentration of PpIX–AgNPs. By following the dual-step irradiation approach described in Scheme S1 (ESI†), a concentration-dependent trend in log reduction within 4 and 24 h post-irradiation time was observed with 1.5 > 1.0 > 0.5 μg mL^−1^. The maximum log reduction achieved at 24 h for the different concentrations (1.5, 1.0, and 0.5 μg mL^−1^) was 6.6 log, 6.1 log, and 4.9 log (Fig. 5b). No significant difference was observed between the inactivation achieved at 1.5 and 1.0 μg mL^−1^, which indicates that lower concentrations can effectively eliminate MRSA. Therefore, our findings demonstrate that by utilizing the dual-step irradiation strategy, the PpIX–AgNPs concentration can be decreased by at least 33.3%, achieving antibacterial efficacy similar to that of single irradiation. Even more relevant, if we compare with the single irradiation method, a reduction in the amount of PpIX–AgNPs of 67% can be achieved. The effective light modulation of this system can have a major effect on decreasing the concentration of nanoparticles used to eliminate MRSA and reduce any potential side effects. In dark conditions, the dual-step irradiation setup at all concentrations shows minimal antibacterial activity (Fig. S13, ESI†). Control experiments with AgNPs of equivalent concentrations were assessed to validate the dual-step irradiation setup (Fig. S14, ESI†). In the absence of light, a minimal inactivation effect is observed for AgNPs. However, under light conditions, like the antibacterial results described above, a log reduction of 3.2, 2.8, and 1.6 was determined at AgNPs concentrations of 0.75, 0.50, and 0.25 μg mL^−1^, respectively, which are the equivalent amount of AgNPs used in PpIX–AgNPs. These values are still significantly lower than those achieved by PpIX–AgNPs. Control experiments with cysPpIX at 1 μM (equivalent to 1.5 μg mL^−1^ PpIX–AgNPs) under dual-step irradiation show up to ∼6 log MRSA inactivation, with no inactivation observed in the absence of light (Fig. S15a, ESI†).

Previous studies have revealed that bacterial culture media negatively impacts the antimicrobial activity of metal ions and nanoparticles.^18,49,63^ In particular, the presence of proteins dramatically decreases the inactivation effect of nanoparticles. Studies have shown that MIC in *E. coli* and *P. aeruginosa* increased by 6–15 fold in protein-rich TSB media than in LB and NB.^18^ In this work, we tested the antibacterial action of PpIX–AgNPs (1.5 μg mL^−1^) against MRSA in NB medium at a concentration of 1.5 μg mL^−1^ under single and dual-irradiation mode. It is important to point out that the performance of the nanoplatform is tested in overgrowth conditions of MRSA with an initial concentration of ∼10^8^ CFU per mL. As seen in Fig. 5c, at this concentration, the PpIX–AgNPs with single and dual-step irradiation modes showed minimal inactivation (<1 log) in NB, which is quite a dramatic reduction compared with the same experiment carried out in DPBS (Fig. 5a). A control experiment in NB using cysPpIX (Fig. S15b, ESI†), shows a ∼5-log MRSA inactivation. This corroborates that the major impact of the protein corona on the performance of PpIX–AgNPs, is associated to Ag^+^ release.^49,63^ To enhance the antimicrobial outcome, we increased the concentration of PpIX–AgNPs to 5.0 μg mL^−1^ and used the dual-irradiation mode. A 1.83, 2.73, and 2.00 log reduction in the MRSA population at 0, 4, and 24 h post-irradiation was determined, which is still very low compared with those results obtained in DPBS. To have a more clinically relevant application of this nanoplatform, we evaluated the system at lower MRSA load.^64^ In addition, most studies demonstrating antibacterial action against MRSA using NPs generally utilize initial bacterial cell density in the range of 10^5^–10^7^ CFU per mL.^28,65–67^ We tested the effect of PpIX–AgNPs (5.0 μg mL^−1^) and dual-irradiation mode with a reduced bacterial load corresponding to ∼10^7^ CFU per mL (Fig. 5d). Under these conditions, the inactivation of MRSA resulted in 2.3, 2.9, and 4.9 log reduction at 0, 4, and 24 h post-irradiation, respectively. These results demonstrated that the antibacterial performance of the light-activated PpIX–AgNP is still relevant for clinical conditions despite the major impact of the protein corona associated with the cell media. Interestingly, the cysPpIX concentration of 3.3 μM (equivalent to 5.0 μg mL^−1^ of PpIX–AgNP) also resulted in a 5-log MRSA inactivation (Fig. S15c, ESI†), most likely due to the increased sensitization of Gram-positive bacteria to PDI.^31,68^

## Conclusions

In this work, we evaluated the impact of cell media on the colloidal stability and release of Ag^+^ from PpIX–AgNPs. Three different media were tested: NB, TSB and LB. The performance of the PpIX–AgNPs was compared with DPBS and water. The nanoparticles showed the best colloidal stability on NB, which was mediated by salt and protein content. We found that for PpIX–AgNPs, media with higher salt content like DPBS and LB afforded poor colloidal stability; in contrast, lower salt content like NB produced good colloidal stability even after 24 h. This behavior is likely due to the presence of highly hydrophobic PpIX molecules on the surface of the AgNPs. The protein corona also helps with the colloidal stability of the PpIX–AgNPs, but plays a minor role. Contrary to colloidal stability, where the salt content has a negative impact, in the case of Ag^+^ release, the higher the salt content, the higher the number of Ag^+^ ions generated at the end of the experiment. Therefore, the release of Ag^+^ followed this trend: DPBS ∼ LB > NB ∼ TSB > water, with NB showing better release performance in the dual-step irradiation setup than TSB. The protein corona again seems to play a minor role in the release of Ag^+^. Considering the pros and cons of the three-cell media on the colloidal stability and Ag^+^ generation, NB was selected to continue with the antibacterial experiments.

The capability of the light-activated PpIX–AgNP platform to generate Ag^+^, which can be controlled not only by the light flux but also by the number of light irradiations, was also evaluated in this work. The release of Ag^+^ was determined in DPBS over up to four cycles of irradiation. The data showed that the amount of Ag^+^ ions generated increased after each irradiation in a stepwise release profile fashion. However, the amount of Ag^+^ released after the third and fourth irradiation cycles had a minor effect on the final amount released. Therefore, it was decided that a dual-step irradiation setup was the optimal setting for the experiments with cell media and bacteria.

Finally, we tested the antimicrobial capacity of the PpIX–AgNPs against MRSA. To determine the impact of cell media on the antibacterial performance of the nanoparticles, experiments in DPBS were performed first. The inhibition of MRSA with log reduction values as high as ∼7 was determined using the colony-count method. The bactericidal performance of the PpIX–AgNPs is concentration-dependent and can be improved by using the dual-step irradiation approach. Optimization of the amount of PpIX–AgNPs used for the elimination of bacteria can be achieved by taking advantage of these results. Antibacterial experiments in NB under overgrowth conditions of MRSA corroborated the significant impact of the cell media on the performance of PpIX–AgNPs. The best conditions optimized using DPBS had a minor inhibition effect on MRSA in the presence of NB. Nevertheless, using clinically relevant MRSA cell density, the PpIX–AgNPs achieved similar inhibition values to those in DPBS. We envision that the light-activated capability of the PpIX–AgNP platform is a promising approach to eliminate ARB.

## Author contributions

Investigation: V. G. and A. J. N., conceptualization: V. G., M. M. and J. V.-E., supervision: M. M. and J. V.-E., funding acquisition: M. M. and J. V.-E., writing – original draft: V. G., writing – review & editing: V. G., A. J. N., M. M. and J. V.-E.

## Conflicts of interest

There are no conflicts to declare.

## Supplementary Material

TB-013-D5TB00081E-s001

## Data Availability

The data supporting this article have been included as part of the ESI.†
