# Supplementary material for: Impact of protein corona and light modulation on the antibacterial activity of light-activated silver nanoparticles
Source: J Mater Chem B. 2025 May 20;13(25):7415–27. doi: 10.1039/d5tb00081e (PMC12121351; doi:10.1039/d5tb00081e)
Supplement: TB-013-D5TB00081E-s001 [file TB-013-D5TB00081E-s001.pdf]

Supplementary Information (SI) for Journal of Materials Chemistry B.
This journal is © The Royal Society of Chemistry 2025

Impact of protein corona and light modulation on the antibacterial activity of light-activated silver nanoparticles

Authors: Varsha Godakhindi [a,b], Anjuman Jannati Nur [c], Mariya Munir [c,d*], Juan L. Vivero-Escoto [a,b,d*]

Corresponding authors: jviveroe@charlotte.edu; mmunir@charlotte.edu

[a]Department of Chemistry, University of North Carolina Charlotte, Charlotte, NC, 28223, USA

[b]Nanoscale Science program, University of North Carolina Charlotte, Charlotte, NC, 28223, USA.

[c]Department of Civil and Environmental Engineering, University of North Carolina Charlotte, Charlotte, NC, 28223, USA

[d]Center for Innovation, Translational Research and Applications of Nanostructured Systems, University of North Carolina Charlotte, Charlotte, NC, 28223, USA

SUPPORTING TABLES

Table S1. TEM size, Hydrodynamic size, and ζ-potential for AgNPs and PpIX-AgNPs in DPBS (1 mM).

Sample Name	TEM size (n= 150) (nm)	Hydrodynamic Size (nm)	ζ-potential (mV)
AgNP	42.2 ± 7.8	37.8 ± 0.3	-47.1 ± 3.6
PpIX-AgNP	42.2 ± 8.9	64.1 ± 0.6	-56.7 ± 3.0

Table S2. Summary of ζ-potential for PpIX-AgNPs in various culture media and DPBS at 0 and 24 hours.

Sample Name	0 hour	24 hours
DPBS (1 mM)	-38.4 ± 0.8 mV	-34.4 ± 4.1
DPBS (10 mM)	-21.4 ± 1.5 mV	-24.1 ± 1.8
NB	-17.8 ± 0.4 mV	-19.1 ± 0.7
TSB	-23.1 ± 1.8 mV	-21.1 ± 1.0
LB	-18.7 ± 1.3 mV	-16.5 ± 1.3

Table S3. Composition of bacterial culture media, adjusted to 1.0 L solution.

Ingredients	NB	Tryptic Soy Broth	Luria Broth	DPBS
Glucose	1 gram	2.5 grams	-	-
Peptone	15 grams	-	-	-
Sodium Chloride	6 grams	5 grams	10 grams	8 grams
Yeast Extract	3 grams	-	5 grams	-
Tryptone (SELECT)	-	17 grams	10 grams	-
Soytone (Soybean)	-	3 grams	-	-
Dipotassium Phosphate	-	2.5 grams	-	0.2 grams
Potassium chloride	-	-	-	0.2 grams
Sodium Phosphate (dibasic)	-	-	-	1.15 grams
Extra components such pH adjuster	pH 7.5 ± 0.2	pH 7.3 ± 0.2	pH 7.0; NaOH	

Table S4. Salt and protein content (in w/v %) in varying media conditions. The % of protein content includes peptone, tryptone, soytone, and yeast extract. In the case of % of the salt content includes sodium chloride, dipotassium phosphate, and potassium chloride.

Culture media	w/v % of salt	w/v % of protein
DPBS	0.95	0.0
NB	0.60	1.8
TSB	0.80	2.0
LB	1.00	1.5

Table S5. ICP-OES digestion results in varying concentrations of PpIX-AgNPs.

Sample Name	Total Ag^+ amount (µg/L)	
	Average	Std Dev
1.5 PpIX-AgNPs	2749.92	45.91
1.0 PpIX-AgNPs	1786.24	195.93
0.5 PpIX-AgNPs	931.56	25.98

Table S6. Kinetic release rates for dual-step irradiation of varying PpIX-AgNP concentration.

Media	Phase	Slope	R2
1.5 ug/mL	Burst_1	21.50	1.00
	Burst_2	7.58	0.95
1.0 ug/mL	Burst_1	18.68	0.99
	Burst_2	9.87	1.00
0.5 ug/mL	Burst_1	12.31	0.99
	Burst_2	7.20	0.98

Table S7. Kinetic release rates for single irradiation in varying culture media.

Media	Phase	Slope	R^2
**Water**	Burst	8.26	0.99
	Steady	0.02	0.90
**DPBS**	Burst	23.66	0.97
	Steady	0.07	0.98
**NB**	Burst	17.83	0.99
	Steady	0.06	0.98
**TSB**	Burst	12.34	0.98
	Steady	0.14	0.89
**LB**	Burst	22.21	0.98
	Steady	0.39	0.99

Table S8. Kinetic release rates for DIR irradiation in varying culture media.

Media	Phase	Slope	R^2
**Water**	Burst_1	4.13	0.92
	Burst_2	3.55	1.00
**DPBS**	Burst_1	9.93	1.00
	Burst_2	6.61	0.82
**NB**	Burst_1	12.59	0.98
	Burst_2	7.79	0.94
**TSB**	Burst_1	7.77	1.00
	Burst_2	6.25	0.99
**LB**	Burst_1	10.64	1.00
	Burst_2	7.79	0.94

SUPPORTING SCHEMES

Scheme S1. Schematic demonstrating the multi-step irradiation setup (MIS) employed for Ag^+ release and bacterial inactivation experiments.

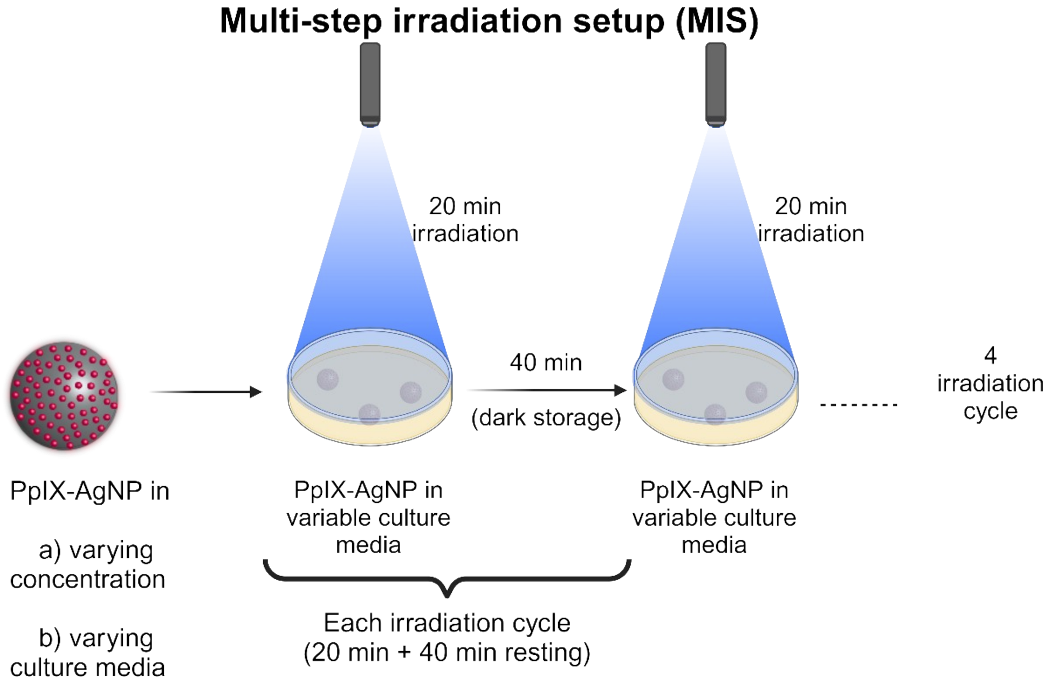

Determination of number of PpIX molecules per silver nanoparticles (AgNP)

By assuming that AgNPs are spherical in shape, the mass of each nanoparticle (m_{AgNP}) was calculated based on average diameter determined from TEM and density value of 8.578 g/mL obtained from Tadjiki et al [[1]]. The number of PpIX molecules per nanoparticle of 241,831 was calculated using the Equation (3).

Equation 3

$$PpIX\ molecules\ per\ AgNP = \frac{m_{AgNP}\, x\, Mf_{(PpIX:AgNP)}\, x\, N_a}{MW_{cysPpIX}}$$

Where, m_{AgNP} is the mass of each AgNP, Mf is mass fraction PpIX:AgNP (0.448:0.552) for PpIX-AgNP, N_a is the Avogadro's constant and $MW_{cysPpIX}$ is the molecular weight of cysPpIX.

SUPPORTING FIGURES

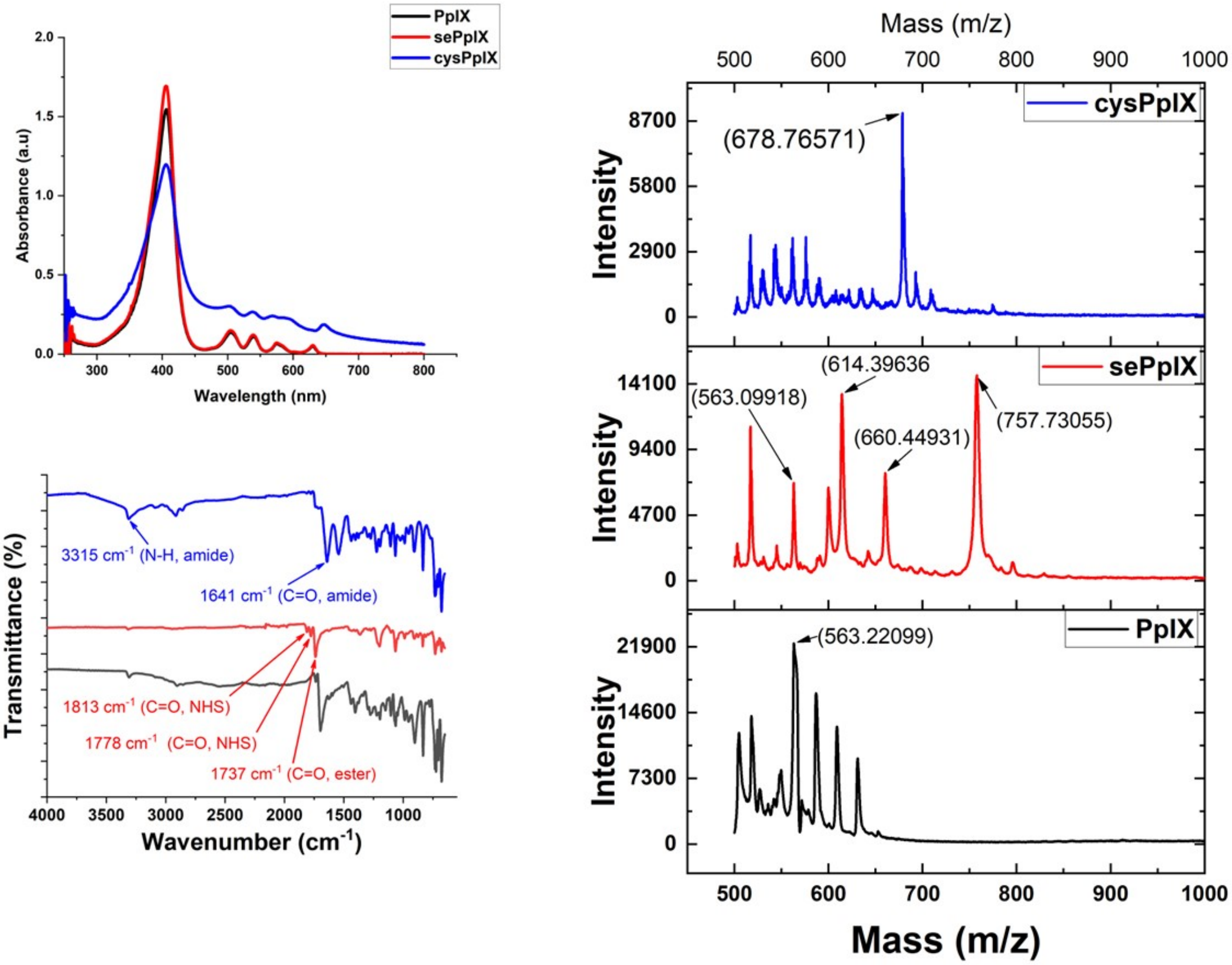

Figure S1. UV-Vis (DMF), FT-IR, MALDI-MS spectra for PpIX (black), sePpIX (red) and cysPpIX (blue). Expected m/z: [PpIX]+ = 562.66 / Observed: m/z: [PpIX+1]+ = 563.22; Expected m/z: [sePpIX]+ = 756.84/Observed: m/z: [sePpIX+1]+ = 757.73; Expected m/z: [cysPpIX]+ = 680.93/ Observed: m/z: [cysPpIX-2]+ = 678.76.

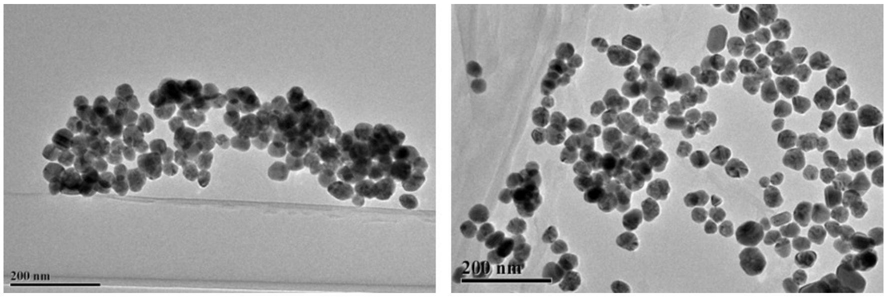

Figure S2. Characterization of AgNPs and PpIX-AgNPs using Transmission electron microscope (TEM) images.

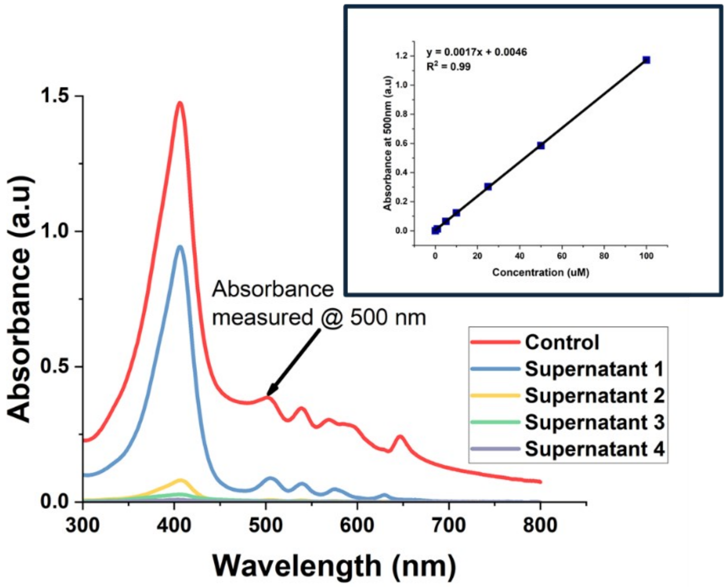

Figure S3. UV-Vis plot for supernatants collected before (control) and after (Supernatant 1-4) the reaction between cysPpIX and AgNPs. (inlet) Calibration curve for the quantification of cysPpIX (generated using absorbance at 500 nM).

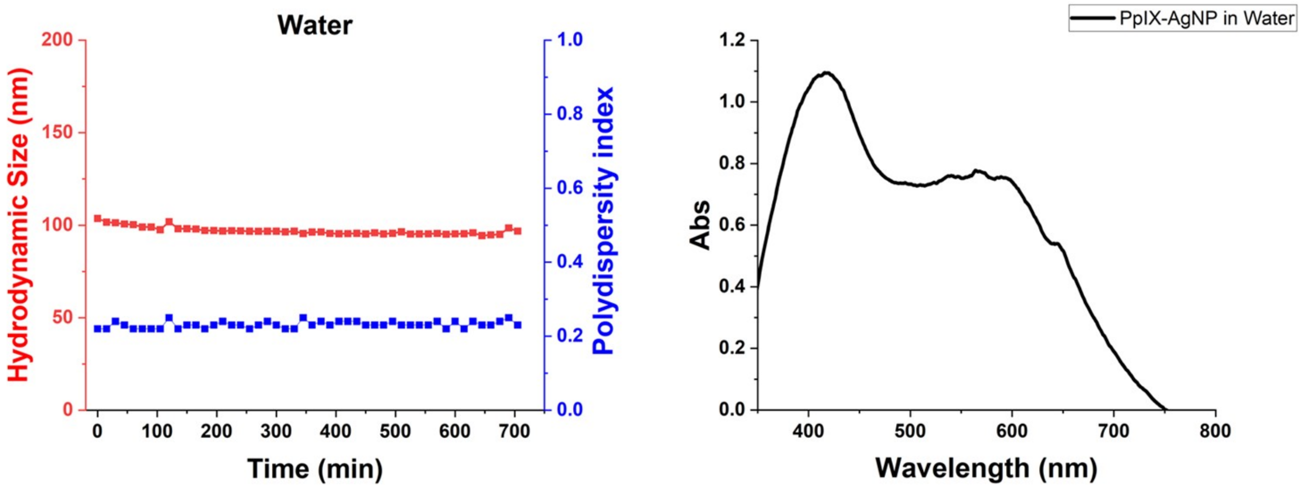

Figure S4. Colloidal stability of PpIX-AgNPs in water. Time-resolved hydrodynamic size and PdI for 12 hours. UV-Vis of PpIX-AgNPs in water (right).

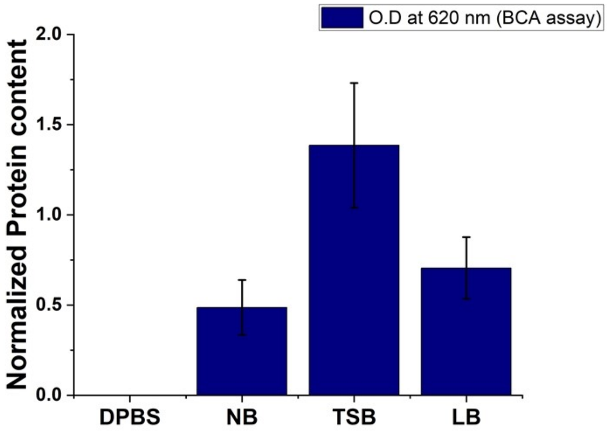

Figure S5. Qualitative assessment of protein content on PpIX-AgNPs surface incubated in bacterial culture conditions post 24-hour incubation calculated using BCA assay.

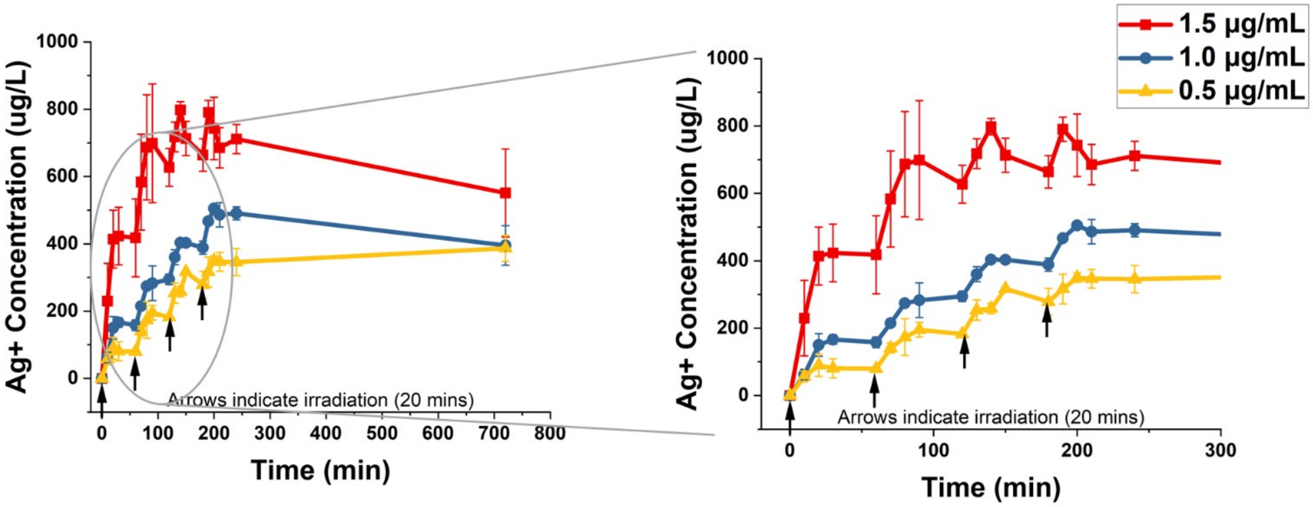

Figure S6. Cumulative Ag^{+} release kinetics for varying concentrations of PpIX-AgNPs under multi-step irradiation (MIS) setup.

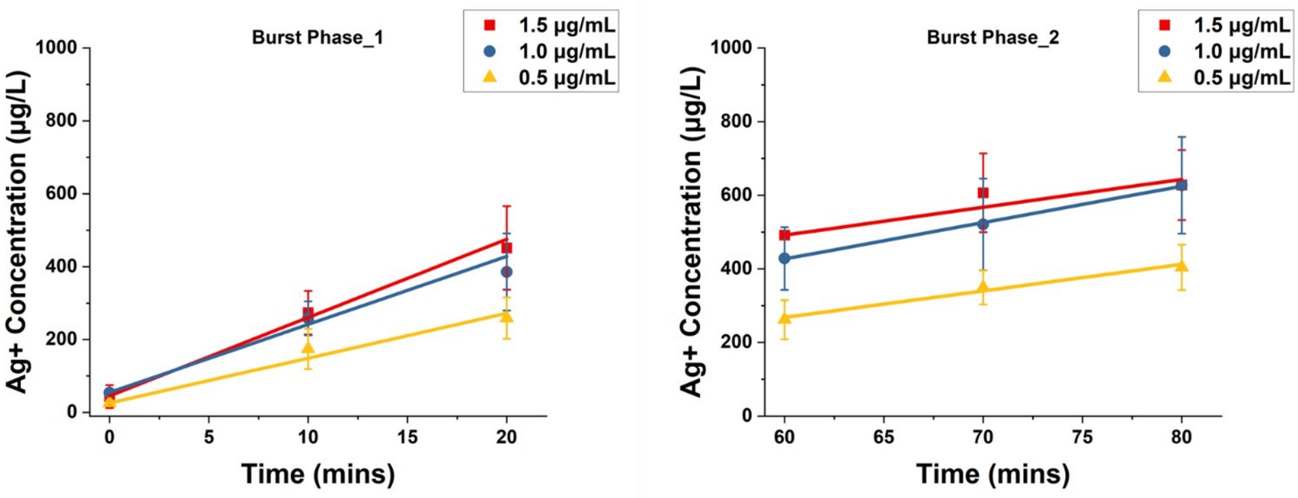

Figure S7. Kinetic release linear fitted plots for varying concentrations of PpIX-AgNPs in DPBS under dual-step irradiation setup.

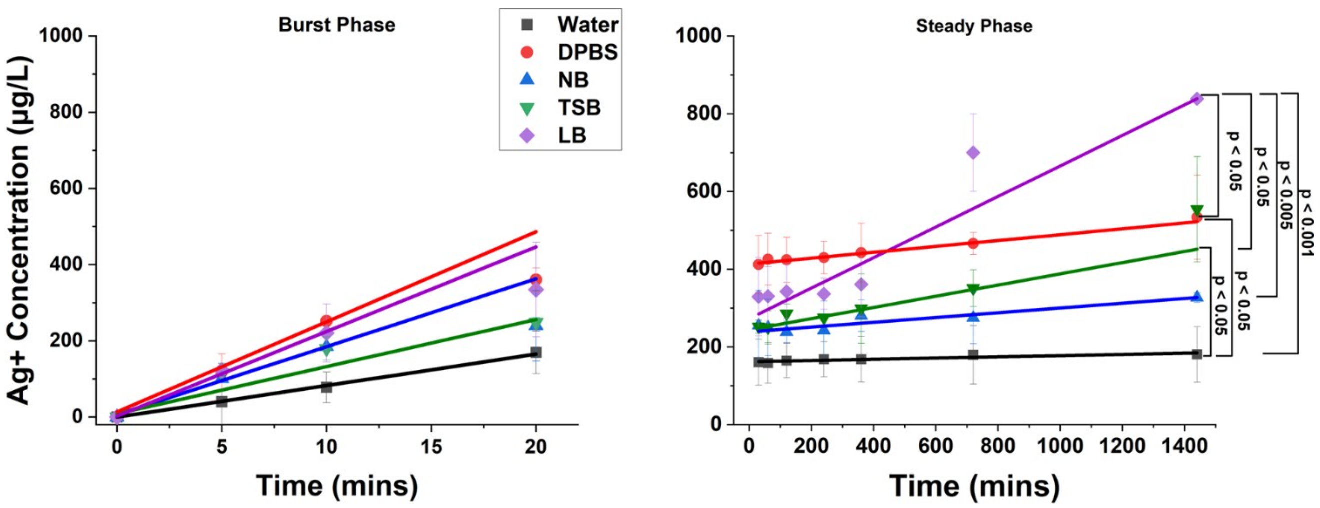

Figure S8. Kinetic release linear fitted plots for PpIX-AgNPs in DPBS and bacterial culture conditions under single irradiation setup.

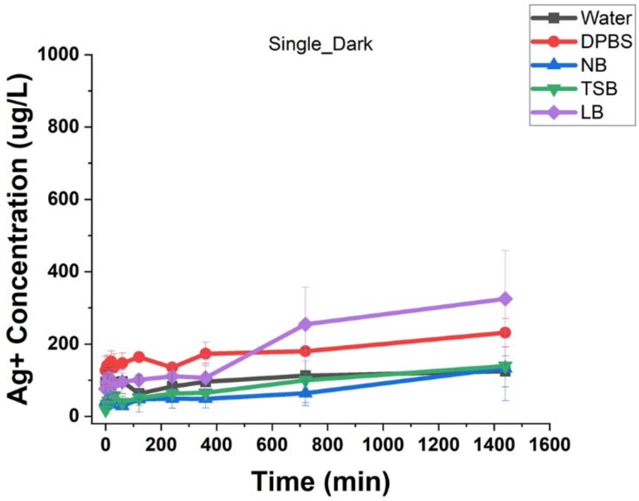

Figure S9. Ag^{+} release kinetics in the absence of light PpIX-AgNPs in DPBS and bacterial culture conditions under the single irradiation setup.

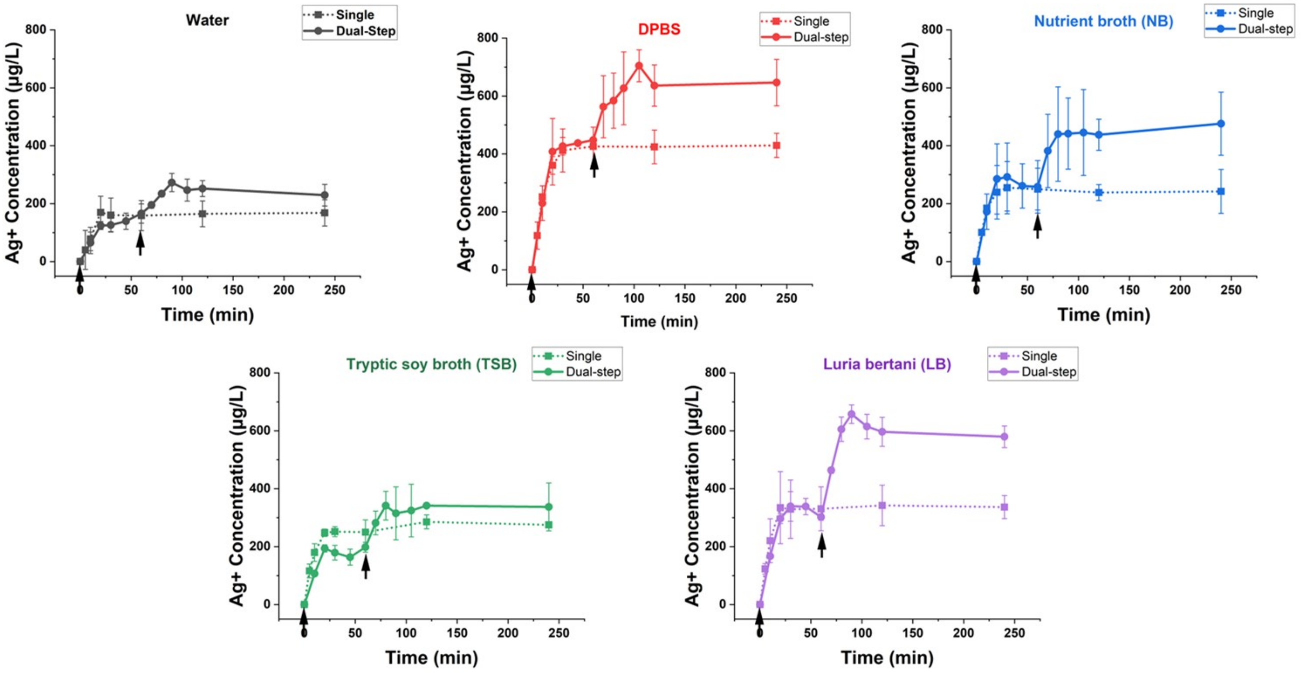

Figure S10. Comparison of Ag^{+} release kinetics for PpIX-AgNPs (1.5 µg/mL) under single and dual-step irradiation setup for water, DPBS and bacterial culture conditions.

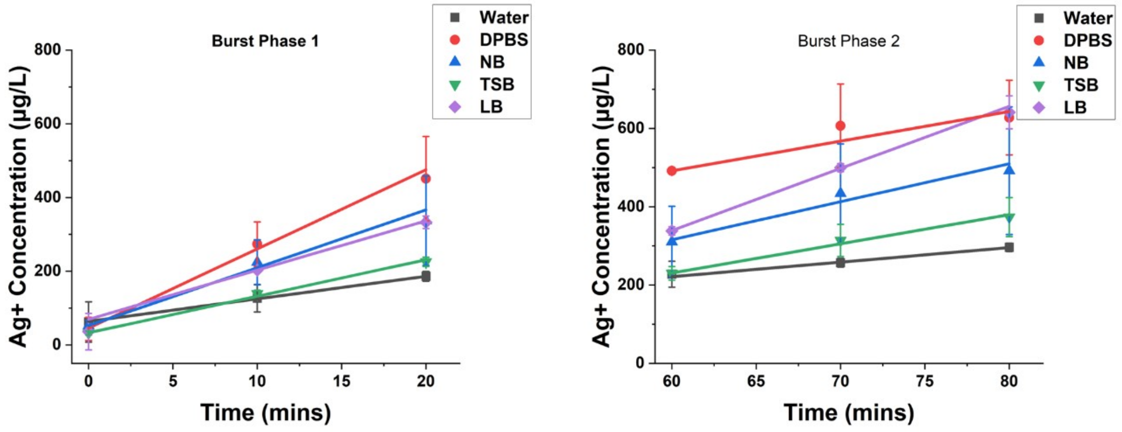

Figure S11. Kinetic release linear fitted plots for PpIX-AgNPs in DPBS and bacterial culture conditions under dual-step irradiation setup.

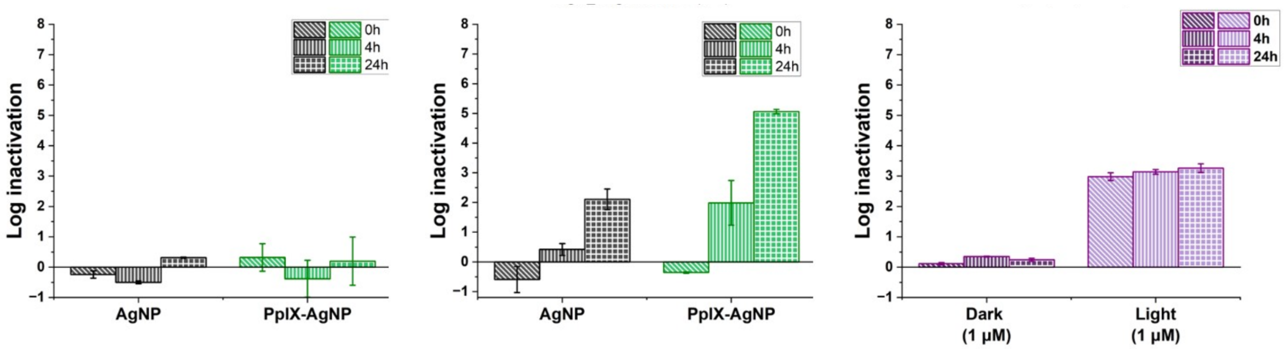

Figure S12. Control experiments in DPBS for MRSA inactivation (single irradiation) using AgNPs and PpIX-AgNPs (1.5 µg/mL) in the absence (left) and presence (center) of light. (right) MRSA inactivation under single irradiation in DPBS using cysPpIX (1 µM) in the absence (Dark) and presence of light (Light).

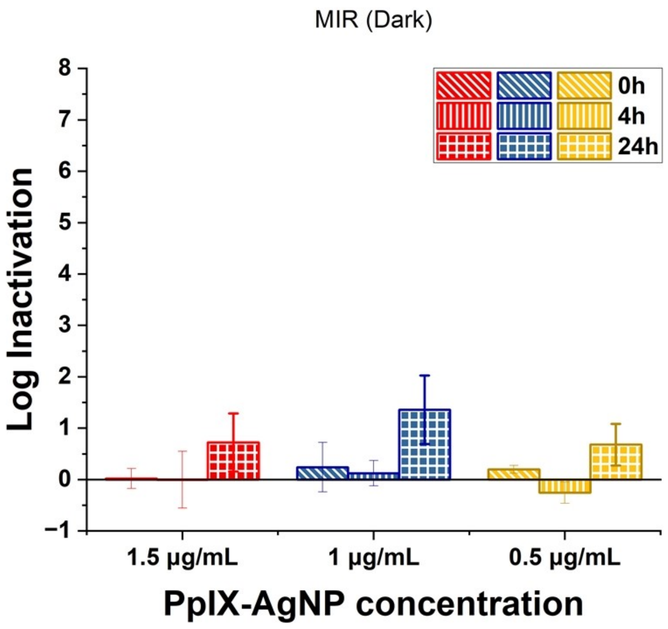

Figure S13. The antibacterial activity of varying concentrations of PpIX-AgNP in DPBS tested in the absence of light.

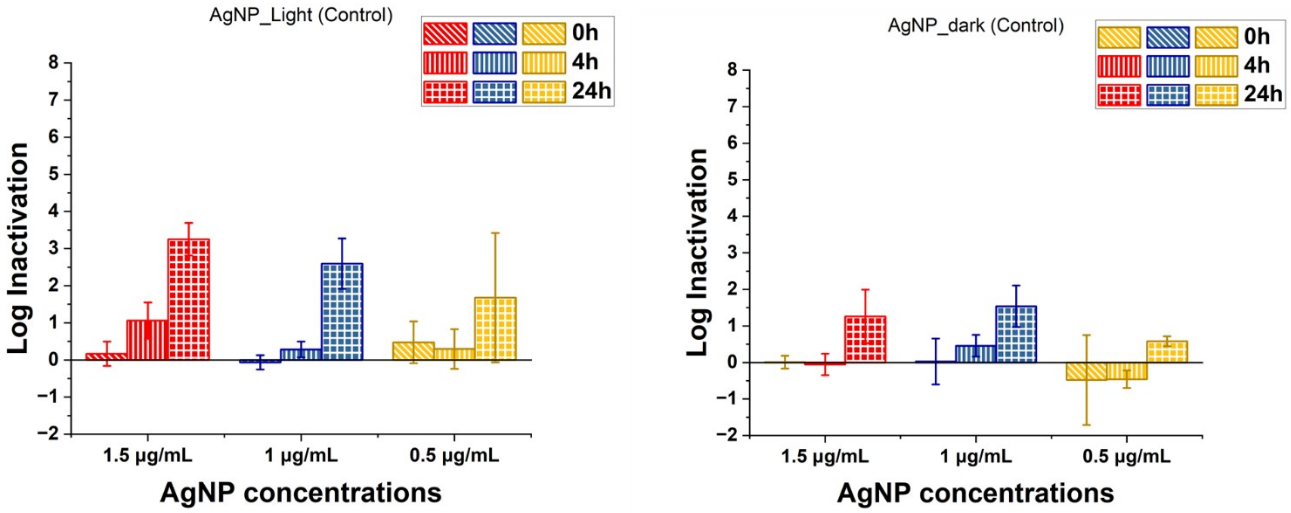

Figure S14. The antibacterial activity of varying concentrations of AgNPs in DPBS tested in the presence (left) and absence of light (right).

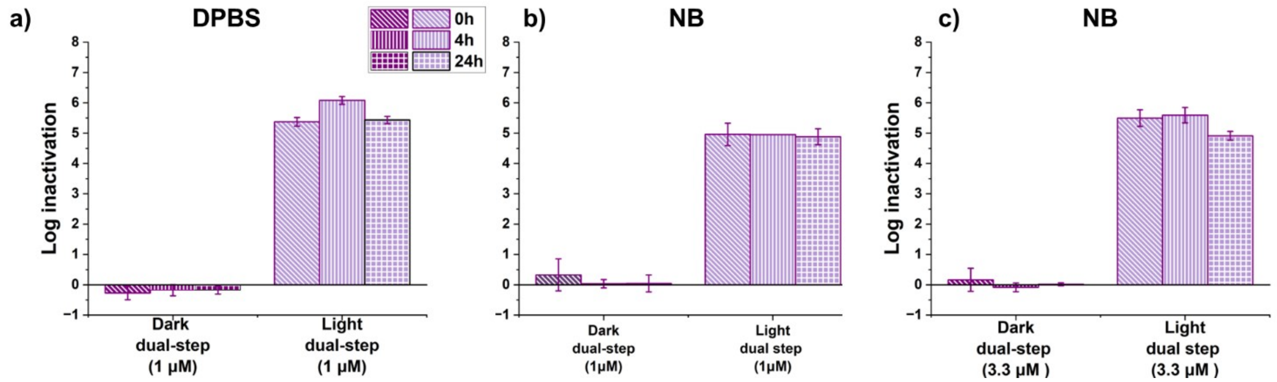

Figure S15. Control experiments using cysPpIX under dual-step irradiation conditions in a) DPBS, b) Nutrient broth (NB) at concentration 1 μM (equivalent to 1.5 μg/mL of PpIX-AgNP), and c) NB at concentration 3.3 μM (equivalent to 5.0 μg/mL of PpIX-AgNP).

1. Tadjiki, S.; Montaño, M. D.; Assemi, S.; Barber, A.; Ranville, J.; Beckett, R., Measurement of the Density of Engineered Silver Nanoparticles Using Centrifugal FFF-TEM and Single Particle ICP-MS. *Analytical Chemistry* **2017,** *89* (11), 6056-6064.
